# Increased Recruitment of Domain-General Neural Networks in Language Processing Following Intensive Language-Action Therapy: fMRI Evidence From People With Chronic Aphasia

**DOI:** 10.1044/2020_AJSLP-19-00150

**Published:** 2020-08-24

**Authors:** Felix R. Dreyer, Lea Doppelbauer, Verena Büscher, Verena Arndt, Benjamin Stahl, Guglielmo Lucchese, Olaf Hauk, Bettina Mohr, Friedemann Pulvermüller

**Affiliations:** a Brain Language Laboratory, Department of Philosophy and Humanities, Freie Universität Berlin, Germany; b Cluster of Excellence Matters of Activity, Image Space Material, Humboldt Universität zu Berlin, Germany; c Einstein Center for Neurosciences Berlin, Germany; d Berlin School of Mind and Brain, Humboldt University Berlin, Germany; e Department of Neurology, University Medicine Greifswald, Germany; f Department of Neurology, Charité Universitätsmedizin Berlin, Germany; g Max Planck Institute for Human Cognitive and Brain Sciences, Leipzig, Germany; h Psychologische Hochschule Berlin, Germany; i Medical Research Council Cognition and Brain Science Unit, Cambridge, United Kingdom; j ZeNIS-Centre for Neuropsychology and Intensive Language Therapy, Berlin, Germany; k Department of Psychiatry, Charité Universitätsmedizin Berlin, Germany

## Abstract

**Purpose:**

This study aimed to provide novel insights into the neural correlates of language improvement following intensive language-action therapy (ILAT; also known as constraint-induced aphasia therapy).

**Method:**

Sixteen people with chronic aphasia underwent clinical aphasia assessment (Aachen Aphasia Test [AAT]), as well as functional magnetic resonance imaging (fMRI), both administered before (T1) and after ILAT (T2). The fMRI task included passive reading of single written words, with hashmark strings as visual baseline.

**Results:**

Behavioral results indicated significant improvements of AAT scores across therapy, and fMRI results showed T2−T1 blood oxygenation-level-dependent (BOLD) signal change in the left precuneus to be modulated by the degree of AAT score increase. Subsequent region-of-interest analysis of this precuneus cluster confirmed a positive correlation of T2−T1 BOLD signal change and improvement on the clinical aphasia test. Similarly, the entire default mode network revealed a positive correlation between T2−T1 BOLD signal change and clinical language improvement.

**Conclusion:**

These results are consistent with a more efficient recruitment of domain-general neural networks in language processing, including those involved in attentional control, following aphasia therapy with ILAT.

**I**ntensive language-action therapy (ILAT), a type of therapy also known as “constraint-induced aphasia therapy” or “constraint-induced language therapy,” has been shown clinically to be an effective method for aphasia therapy at the chronic stage in a number of randomized controlled trials (RCTs; Berthier et al., 2009; Heikkinen et al., 2019; Kurland et al., 2016; Pulvermüller et al., 2001; Stahl et al., 2018; Stahl et al., 2016; Szaflarski et al., 2015). Recent evidence suggests that a moderately intensive delivery of at least 6 hr/week is important for its success, whereas further increase in intensity (Stahl et al., 2018) and the application of certain constraints, including the exclusion of gestures (Kurland et al., 2016), apparently come without additional added value. A crucial feature of ILAT appears to be the embedding of language in social communicative action, as shown by Stahl et al. (2016). This study presented a cross-over RCT trial with all patients performing both ILAT and an intensive naming therapy for 2 weeks each. In the ILAT condition, patients were asked to request objects and respond to request in a card game context in group therapy sessions, whereas they were asked to name and describe counterbalanced objects in an intensive naming therapy. Although therapy intensity, stimulus materials, and linguistic target forms were the same, both therapy conditions differed in their efficiency, as demonstrated by a significant cross-over interaction between therapy type and time that showed consistent improvement only for ILAT, but not for the naming therapy. Because of the documented effects of both intensity and embedding of language into action and social interaction, we believe that the term “intensive language-action therapy” (ILAT rather than “constraint-induced aphasia therapy” or “constraint-induced language therapy”) might represent the most pertinent label for this kind of therapy.

Research on the neural mechanisms underlying ILAT-induced language improvements indicates the relevance of different types of neuroplastic processes. Meinzer et al. (2008) investigated a cohort of 11 people with chronic aphasia and showed that, following a 2-week interval of constraint-induced aphasia therapy, language improvements correlated positively with functional magnetic resonance imaging (fMRI) blood oxygenation-level-dependent (BOLD) signal increase (elicited in a picture-naming paradigm) in individual perilesional regions of the left hemisphere. However, other research pointed toward a contribution of right-hemispheric homotops of left perisylvian language areas. Mohr et al. (2014) reported neurometabolic activity of six people with aphasia, who showed not only significant language improvements after a 2-week interval of ILAT but also significant fMRI BOLD signal increase in areas homotopic to the lesion, that is, in right perisylvian inferior frontal and temporal areas, in a sentence comprehension paradigm. This observation of a specific role of the right hemisphere is in line with earlier magnetoencephalography findings (Breier et al., 2007) on ILAT effects. In addition, partly opposing findings on the role of the right hemisphere were reported by Richter et al. (2008). Here, right-hemispheric fMRI BOLD signal decrease, rather than signal increase, was found to correlate positively with clinical language improvement in a cohort of 16 people with chronic aphasia, following a 2-week interval of ILAT. However, at the same time, initial right-hemispheric activation correlated with subsequent therapy success in this sample. Furthermore, other studies provide results supporting an involvement of both left- and right-hemispheric areas in ILAT-driven aphasia recovery (Breier et al., 2006, 2007, 2009; Lucchese et al., 2017; Mohr et al., 2016).

These diverging results on the neural substrates of ILAT-induced language plasticity in chronic aphasia parallel those on the neural mechanisms supporting therapy-related aphasia recovery in general, independent of exact therapy type and characteristics. In recent reviews (e.g., Crinion & Leff, 2015; Hartwigsen & Saur, 2019; Mohr, 2017; Turkeltaub et al., 2011), recruitment of left-hemispheric extra- and perilesional, as well as right-hemispheric areas homotopic to lesioned left perisylvian language areas, is reported to support aphasia recovery, thus mirroring the aforementioned ILAT-specific results. In addition, Hartwigsen and Saur (2019) presented a further neural (and cognitive) mechanism in their review that may support therapy-related language recovery: A more efficient recruitment of domain-general cognitive resources and the corresponding neural substrates in linguistic processing. Earlier findings indicate a close coupling of linguistic and domain-general cognitive systems in healthy populations (e.g., Rodd et al., 2010; Vaden et al., 2013) and a spatial overlap of domain-general and language-specific processing networks in frontal and parietal areas (Geranmayeh, Wise, et al., 2014; Piai, Roelofs, Acheson, & Takashima, 2013). Furthermore, recent results from Sliwinska et al. (2017) show that transcranial magnetic stimulation of domain-general frontal midline cortex can improve behavioral performance in a (pseudo-language) vocabulary learning task in healthy participants. Therefore, it appears to be not unlikely that domain-general systems may contribute to language recovery in people with aphasia (for an extensive review, see also Geranmayeh, Brownsett, et al., 2014). Evidence for this position can be seen, for example, in the work of Brownsett et al. (2014). This study reported fMRI BOLD activity in a speech listening task in the midline frontal cortex to correlate positively with communicative abilities in chronic aphasia. This area was interpreted to be part of the salience network (SN; Seeley et al., 2007), which is involved in attentional control processes and shows anticorrelated activation patterns to the default mode network (DMN), associated to idle or passive states (Raichle et al., 2001). Further evidence for an involvement of domain-general systems in language recovery in aphasia can be seen in the results by Geranmayeh et al. (2017). This study found signal increase in presupplementary motor area and dorsal anterior cingulate, two areas assumed to be involved in domain-general processing networks, to correlate positively with spontaneous language recovery in the first 4 months after stroke.

The current study aimed to contribute to the discussion on which brain areas are involved in clinical language improvements across ILAT, with a special focus on contributions of domain-general systems, by investigating a cohort of 16 people with chronic poststroke aphasia and comparing their clinical aphasia measures, as well as fMRI correlates of linguistic processing, before and after the administration of ILAT. The current study applied a passive reading task without any response requirements. This choice was made to exclude any response-related biases while at the same time engaging relevant attentional and executive processes necessary for language perception and understanding. The analyses were data driven, exploring possible correlations between therapy-related improvements in clinical language performance and metabolic changes.

## Method

### Patients

**Figure 1:**
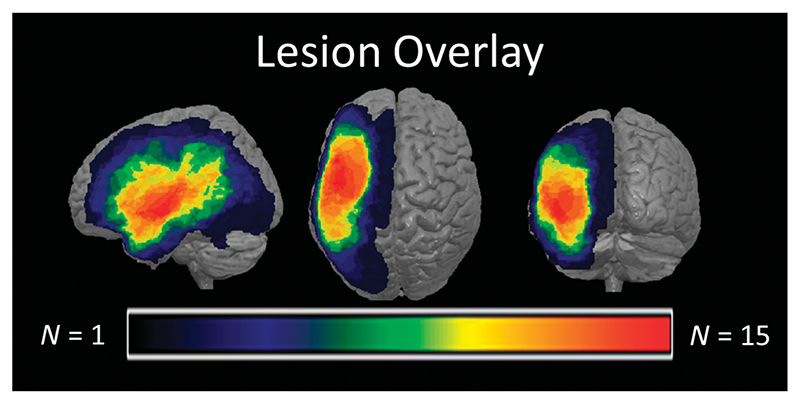
Lesion overlay map of patients included in the analysis. Lesions are mapped on MNI standard space. Warmer colors depict higher lesion overlap. MNI = Montréal Neurological Institute.

In total, 48 people with chronic aphasia following left-hemispheric stroke participated in ILAT therapy. A subset of 24 people with aphasia fulfilled MRI inclusion criteria and was scanned before the start and 2−4 weeks later, after the end of therapy. Data of eight individuals were excluded due to artifacts during scanning (excessive movements and/or pronounced fatigue during scanning). Patient and therapy characteristics of the remaining 16 people with aphasia included in the analysis can be found in Table 1. A lesion overlay of this sample is presented in Figure 1, with largest lesion overlap (*N* = 15) in the left superior temporal cortex and the left insula. On average, people with aphasia were 55.9 years of age (*SE* = 4.2 years) and started ILAT at 72 months poststroke on average (*SE* = 18.3), and eight people with aphasia were female. Results of the Aachen Aphasia Test (AAT; Huber et al., 1983, 1984) classified 11 people with aphasia to suffer from Broca’s aphasia. The remaining patients were diagnosed as global (two), Wernicke (one), and amnesic (one) people with aphasia, whereas one aphasia was nonclassifiable. Informed consent was obtained from all patients, and procedures were approved by the ethics committee of the Charité University Hospital in Berlin, Germany.

### Treatment Protocols

Patients from two different RCTs addressing the effect of ILAT at different intensities and durations (Stahl et al., 2018) and in comparison to other intensive speech-language therapies (German Clinical Trials Register, identifier: DRKS00007829) were pooled in this analysis. The reason for this was the relatively low percentage with which patients were available for fMRI scanning. Therefore, the total therapy duration was either 24 hr with an intensity of 6 hr/week, 25 hr with an intensity of 12.5 hr/ week, or 48 hr with an intensity of 12 hr/week. Due to small sample sizes in individual duration and intensity conditions, results from all studies were collapsed for analysis.

Exact details of the treatment protocol can be found in Stahl et al. (2018, 2016). In essence, each therapy session had three patients and a therapist seated around a table to engage in language games (see also Difrancesco et al., 2012; Pulvermüller, 1990, 2001) involving matched card pairs. In these games, each player was given a set of picture cards, depicting either objects or actions. Each card had a duplicate that was owned by one of the other players. In one variant of these games, the “request game,” players were asked to request these card duplicates from the other players or to respond to the request of another player. In case a requested duplicate was available, the addressed player handed over the corresponding card to the requesting player. In case the requested card was not available, the addressee rejected the request. The complexity of requests and responses was adjusted to the patients’ individual aphasia severity by varying the difficulty level of target words and required sentence structures.

### Patient Assessment

#### Clinical Aphasia Testing

Aphasic syndromes and severity of the patients was evaluated using the AAT, a standardized German aphasia test battery (Huber et al., 1983, 1984). The following sub-tests of the AAT were used to assess language functions: the Token Test, Verbal Repetition, Naming, and Comprehension. AAT assessments were conducted at baseline, before the start (T1), and after the end of the ILAT interval (T2).

#### fMRI Paradigm

In addition to the AAT, patients participated in an fMRI paradigm at T1 and T2, on the day following clinical assessment. In this paradigm, patients were asked to passively and silently read single words and to avoid any movements during scanning. All stimuli appeared one by one in the middle of a display. To give patients enough time to process words comfortably, individual trials were 2,250−2,650 ms long. To discourage eye movements, we used tachistoscopic word presentation (for 150 ms), with interstimulus intervals randomly varying between 2,100 and 2,500 ms (2,350 ms on average) in which a fixation cross was presented on screen. Tachistoscopic presentation of single words with similar or even shorter presentation times than in the current paradigm has previously been used for studying word processing in people with poststroke aphasia (Neininger & Pulvermüller, 2003; Pulvermüller et al., 2005) or brain tumor (Dreyer et al., 2015; Dreyer et al., 2020), for example, in lexical decision experiments.

A passive reading task was chosen to diminish the influence of nonlinguistic executive and attentional demands, otherwise required in response selection, preparation, and execution of explicit tasks, on fMRI results and to avoid movement related artifacts. Stimulation parameters were similar to an earlier passive reading fMRI study (Dreyer & Pulvermüller, 2018) in terms of trial structure, presentation times, interstimulus interval, and stimuli size. Stimuli consisted of 176 German words and 88 hashmark strings, which served as a visual baseline. Word stimuli were composed of nouns and verbs of abstract or concrete semantics, with an average word length of 7.2 characters (*SD* = 1.7), 2.3 syllables (*SD* =.4), and lemma frequency of 8.5 per million. according to measures taken from the dlex corpus (Heister et al., 2011). Lengths of words and hashmark strings were matched in terms of number of characters (*p* =.89) to avoid basal visual differences between both stimulus types. All stimuli were printed in white uppercase letters on black background, using monospaced Courier New font, and were spanning a maximum of 2° horizontal and 0.6° vertical visual degree.

The reading paradigm performed in the scanner was divided into two blocks of 8 min. Each block started and ended with a baseline phase of 15 s, in which a central fixation cross was presented. Following the last reading block, a T1-weighted MPRAGE scan was collected for approximately 5 min while the patients lay still with their eyes closed to obtain a structural MRI at grain size 1-mm^3^ voxels. During the reading task, a video camera was used to monitor patients’ task compliance.

### Analysis

#### AAT Analysis

Raw AAT performance scores for each subtest were determined, converted into age-normalized standard *T* scores, according to the tests’ instructions, and subsequently averaged across subscales for each patient and time point. Furthermore, differences of average AAT *T* scores between time points were calculated for every patient individually. In addition, results between time points were compared using a paired-samples *t* test on the group level.

#### MRI Lesion Mapping

Lesion templates were created manually for each individual patient on T1 structural MRI images from the first scanning session in MRIcron (www.mricro.com/mricron). Resulting lesion maps were used for lesion cost function masking (Brett et al., 2001), to normalize patients’ structural images and corresponding lesion templates to a Montréal Neurological Institute (MNI) standard space using the Clinical toolbox (Rorden et al., 2012) of the Statistical Parametric Mapping software (SPM8, Wellcome Department of Cognitive Neurology).

#### fMRI Preprocessing

Scanning was performed with a 3-T Siemens Tim Trio magnetic resonance device using a 12-channel head coil. Functional images were acquired in echo-planar sessions with a time repetition of 2,000 ms, a time echo of 30 ms, and a flip angle of 78°. Scans consisted of 32 slices, acquired in descending order, with a voxel size of 3 × 3 × 3 mm and an interslice distance of 0.75 mm. Data preprocessing and analysis were performed using SPM8 (Wellcome Department of Imaging Neuroscience). Before analysis, images were corrected for slice timing and realigned to the first image using sinc interpolation. As a next step, echo-planar images were coregistered to the structural T1 images and were then normalized to MNI using the transformation parameters derived from lesion normalization (see above). Images were resampled with an interpolated spatial resolution of 2 × 2 × 2 mm and spatially smoothed with an 8-mm full-width half-maximum Gaussian kernel. First- and second-level general linear models were derived on the basis of the canonical hemodynamic response function, using a high-pass filter of 128 s to reduce low-frequency noise. In the first-level models, stimulus-type regressors for proper words and hashmarks were included in addition to six regressors accounting for patient movements and head rotations, separately for both blocks of the reading paradigm.

#### fMRI Analysis

In first-level analysis, contrast of words versus visual baseline was determined for each patient and time point individually, thus reflecting the degree of linguistic processing during word reading. To increase statistical power of the analysis, white matter was excluded from the analysis by applying an explicit gray matter mask derived from the gray matter template provided by SPM (binarized by selecting only voxels with a gray matter probability >.5). Furthermore, also the individual normalized patient lesions were masked out in first-level analysis. For second-level analysis, difference images between time points of contrast images for the words versus baseline comparison were calculated (both T2−T1 and T1−T2) for each patient. Subsequently, the resulting difference images were included in a second-level one-sample *t* test, using the behavioral AAT differences between time points as continuous regressors. Analysis of these regressors allowed to identify those voxels that have their T2−T1 and T1−T2 differences in the contrast of words versus visual baseline modulated by clinical language improvement. Significant clusters in this analysis (with voxelwise *p* <.005 and cluster size *k* > 100) were chosen for further region-of-interest (ROI) analysis to directly assess correlations between signal change and changes on behavioral aphasia measures over time. Although we note that this strategy is a case of double dipping, we emphasize that the additional results obtained from the correlation analyses, in particular, sign and magnitude of *r* values, appear as useful complementary information. Here, ROIs were created, and data from the words versus baseline contrast were extracted for each time point using the Marsbar tool-box (Brett et al., 2002) in SPM8 and subsequently subtracted from another. Definition of further anatomical post hoc ROIs was based on the automated anatomical labeling template (Tzourio-Mazoyer et al., 2002), provided in the Wake Forest University pick atlas (Maldjian et al., 2003). All ROIs applied in analysis had no lesions present in the patients under investigation. Furthermore, control analyses were applied, using ILAT intensity and total duration as sole continuous regressors in the T2−T1 and T1−T2 differences analyses for the contrast of words versus visual baseline, in order to identify the influence of ILAT intensity and duration on signal change in isolation. Both variables were also included as covariates in whole-brain models and subsequent ROI correlation analyses. In order to account for the reduction in power following the inclusion of additional covariates, the cluster size threshold was lowered to *k* >40 for the whole-brain analyses. In addition, further second-level analyses were performed in contrasts between words and visual baseline for T1 and T2, as well as for the comparison of this contrast between time points.

## Results

### Behavioral Results

**Figure 2:**
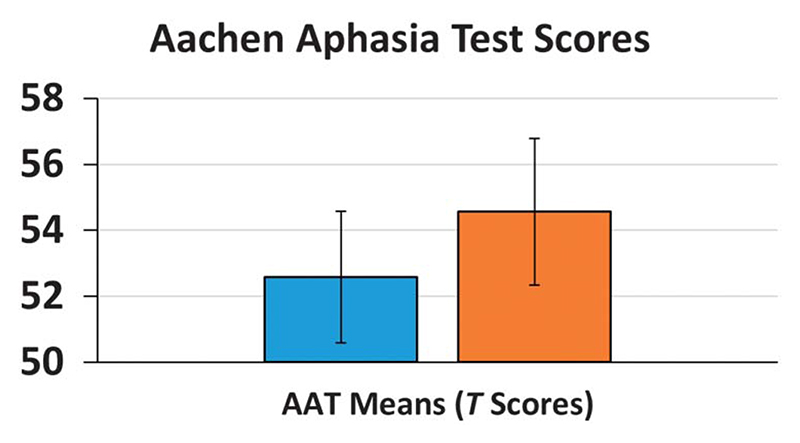
Average Aachen Aphasia Test (AAT) *T* scores before (T1) and after ILAT (T2). Error bars depict the standard error of the mean.

Comparison of mean AAT scores (including all afore-mentioned subtests) before and after therapy revealed a significant clinical language improvement following ILAT, *t*(15) =4.31, *p* <.001 (see Figure 2), with an average *T*-score increase of 2.05 (*SE* = 0.48). Individual AAT scores and differences between time points are summarized in Table 2.

### fMRI Results

**Figure 3:**
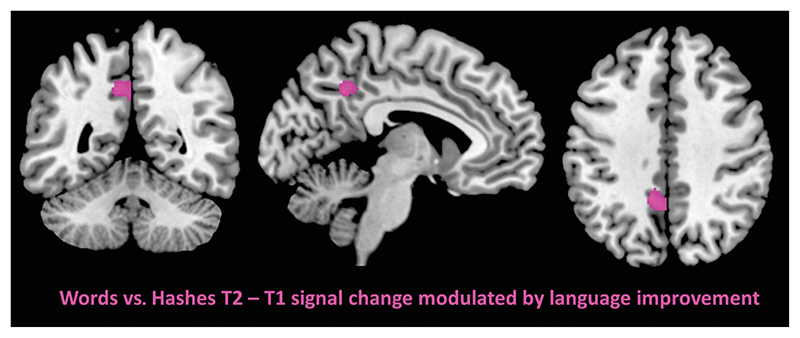
Significant cluster in the left precuneus for the effect of Aachen Aphasia Test increase on signal increase for the contrast words versus visual baseline after intensive language-action therapy at *p* <.005, with a minimum cluster extend *k* =100.

Whole-brain analysis of the regressor effect of the AAT *T*-score T2−T1 change on the T2−T1 signal difference for the contrast of words versus visual baseline revealed a significant cluster in the left precuneus (MNI *xyz* = −10, −48, 40), thresholded at *p* <.005 (uncorrected) and a minimum cluster size of *k* > 100 (see Figure 3 and Table 3). The reverse analysis on signal decrease after therapy (T1−T2) failed to yield significant clusters (using the same voxel- and cluster-wise thresholds). Also, the control analyses using ILAT intensity and ILAT duration as sole continuous regressors did not reveal significant clusters. With the inclusion of either ILAT intensity or duration as covariates, an effect for the AAT T-score T2−T1 change regressor on the T2−T1 signal difference for the contrast of words versus visual baseline was again observed in the left precuneus (see Table S3 in Supplemental Material S1). Results for analyses of simple contrasts of words versus visual baseline for each time point are presented in Table S1 in Supplemental Material S1, and for the comparisons between time points, see Table S2 in Supplemental Material S1.

The significant precuneus cluster in the T2−T1 signal change analysis did not survive family-wise error or false discovery rate correction at a peak- or cluster-wise threshold of *p* <.05; the whole-brain analysis should therefore not be used for strong inferential purposes.

**Figure 4:**
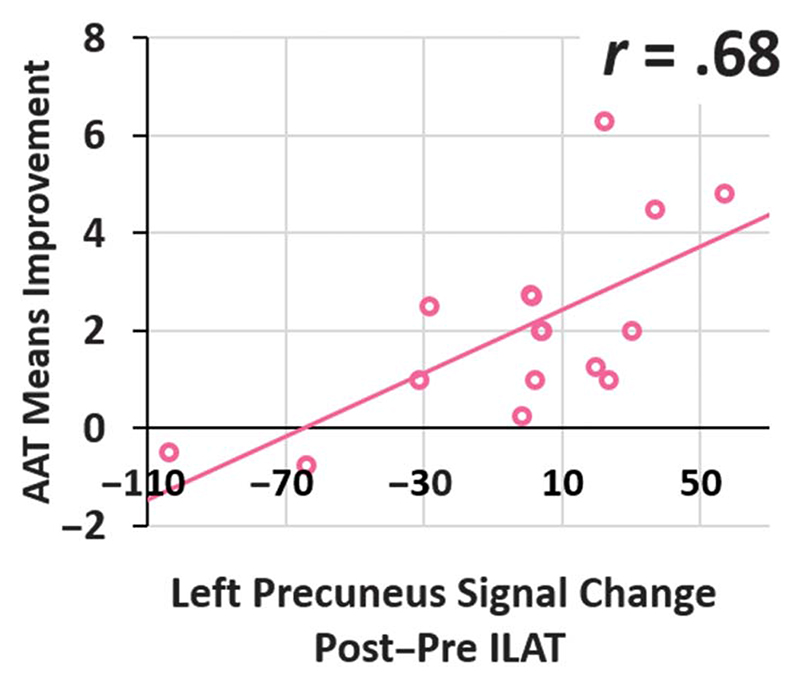
Scatter plot of left precuneus words versus hashmarks T2−T1 blood oxygenation-level-dependent signal change and Aachen Aphasia Test (AAT) results T2−T1. Circles depict data from individual patients, and the linear trendline is represented via the solid line. ILAT = intensive language-action therapy.

However, this finding motivated a set of data-driven post hoc ROI correlation analyses. The first revealed T2−T1 signal change in the left-hemispheric precuneus cluster to correlate positively with AAT improvements (Pearson *r* =.68, *p* =.004; see Figure 4), and this correlation remained significant when ILAT intensity (Pearson *r* =.66, *p* =.007) or total duration (Pearson *r* =.66, *p* =.008) was partialed out (for individual signal strength at each time point per patient, see Figure S2 in Supplemental Material S1). Please note that these analyses should not be interpreted to be separate from the result of the whole-brain analysis reported above, as this would resemble “double-dipping” in analysis (Kriegeskorte et al., 2009), but rather reveals additional information about the nature of the aforementioned finding, as it provides direct measures of correlation strength and direction.

In light of the significant cluster in the precuneus, which is considered to be part of the domain-general DMN (Utevsky et al., 2014), a post hoc ROI of the DMN was defined. This ROI consisted of gray matter components of medial prefrontal and posterior cingulate cortex, lateral temporal cortex, and hippocampal formations, in addition to the precunei of both hemispheres, as those regions were previously discussed to constitute the core regions of the DMN (Buckner et al., 2008). As patients frequently showed lesions in some of these regions, only those voxels were considered for ROI creation that did not show a lesion in any of the patients (please see Figure S5 in Supplemental Material S1 for details).

A further post hoc ROI was set on bilateral superior frontal cortex and the anterior cingulum in order to test for a replication of previous results by Brownsett et al. (2014). This study reported a contribution of the SN (Seeley et al., 2007), a domain-general system for attentional control and anticorrelated functional counterpart of the DMN (Raichle et al., 2001), to language performance in chronic aphasia. In order to further investigate this apparent contrast to the current results, T2−T1 fMRI signal change in both post hoc ROIs was considered for correlation analysis with AAT T2−T1 changes.

**Figure 5:**
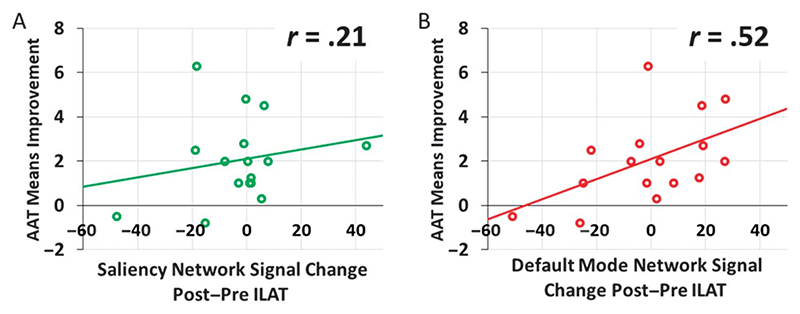
Scatter plot of words versus hashmarks T2−T1 blood oxygenation-level-dependent signal change and Aachen Aphasia Test (AAT) results T2−T1 in the salience network (A, printed in green) and in the default mode network (B, printed in red). Circles depict data from individual patients, and the linear trendlines are represented via the solid line. ILAT = intensive language-action therapy.

Results indicated no significant correlation between the T2−T1 BOLD signal change for the words versus visual baseline contrast in the SN ROI and clinical aphasia improvement (Pearson *r* =.21, *p* =.44; see Figure 5A), but this correlation was significant for the signal change in the DMN ROI (Pearson *r* =.52, *p* =.038; see Figure 5B). The latter correlation remained significant when patient age, years of education, and ILAT intensity (Pearson *r* =.61, *p* =.026) or total duration (Pearson *r* =.55, *p* =.049) were partialed out. For an overview of individual signal strength at each time point per patient in both ROIs, see Figures S3 and S4 in Supplemental Material S1.

The inclusion of the significant left precuneus cluster from the whole-brain analysis in the DMN ROI may compromise the interpretability of results due to double-dipping in the analysis pathway. Therefore, a further control analysis was applied, excluding the significant left precuneus cluster from the ROI definition of the DMN. Results of this control analysis confirmed a significant positive correlation between signal change in the remaining parts of the DMN and clinical aphasia improvement (Pearson *r* =.51, *p* =.041; see also Figure S1 in Supplemental Material S1).

## Discussion

This study investigated the relationship between changes in neurometabolic activity and clinical language performance over a short interval of intensive speech-language therapy with ILAT in a cohort of 16 people with chronic poststroke aphasia. At the behavioral level, patients presented a significant increase of AAT scores following ILAT, in a range comparable to previous studies on ILAT efficiency (Pulvermüller et al., 2001; Stahl et al., 2018, 2016). Regarding the neural substrate underlying this language improvement, significant positive correlations to fMRI BOLD signal strength increase of the contrast words versus visual baseline after ILAT were found in the left precuneus and DMN network ROIs. As the DMN is known to be most active when subjects let their minds wander or think about or evaluate past or future events but do not focus their attention on a specific perceptual task, the stronger activation observed across ILAT therapy in the attention-demanding reading task may seem difficult to explain. However, to solve this riddle, we suggest that, as one possibility, they may just have changed their strategy by adding a grain of “mind wondering” to the reading exercise and putatively to other linguistic tasks, too. What is required when attempting reading a letter with reduced linguistic ability is the “wandering” of the language machinery to a range of alternative lexical items possibly fitting the target string. Such facilitated multiple lexical access and reduced lexical inhibition in reading and in other linguistic tasks may be one possible reason why our patients’ clinical language skills improved, along with an activation increase of the DMN in a passive word reading task.

However, we do not wish to defend this interpretation against alternative views. In line with previous observations, our present results may be interpreted in terms of a relatively more efficient allocation of attentional resources (Brownsett et al., 2014) to language processing in patients with strong improvements in clinical language test performance. Furthermore, previously reported correlations of precuneus activation increase and clinical language improvement following language therapy can be interpreted in terms of improved memory competence (Musso et al., 1999) or better retrieval of episodic memory during language processing (Fridriksson et al., 2007). We note that, in the field of dyslexia research, previous work had already hinted a specific involvement of the left precuneus during successful reading therapy (Krafnick et al., 2011). In this study, a voxel-based morphometry analysis revealed gray matter volume in the left precuneus (among others) to be increased in developmentally dyslexic children following a few weeks of reading intervention. ILAT, as it was conducted with the current patient cohort, does not involve pronounced training of reading (as picture cards are used in therapy), but the precuneus involvement could still, in theory, indicate a carryover effect between linguistic modalities. However, we rather suggest that, in both people with dyslexia and people with aphasia, neurometabolic activity increase in the precuneus indexes improved allocation of general cognitive resources to linguistic processing in general, independent of language modality.

Contrasting to what may be expected following the findings of Brownsett et al. (2014), the DMN, rather than the SN, was observed to show significant activation increase in the course of ILAT correlating with clinical language recovery. This might appear counterintuitive, as activity in the DMN is classically associated with passive or idle brain states, whereas the SN shows activation in cognitive tasks instead (Greicius et al., 2003; Raichle et al., 2001). Activation in both networks has been shown to be negatively correlated, and the amount of deactivation and activation of the DMN (and vice versa the SN) has been demonstrated to be modulated by task difficulty and cognitive affordance (Greicius et al., 2003). Therefore, the increased activation of the precuneus and the broader DMN may be an indication for a decrease of cognitive affordance and a more efficient recruitment of attentional resources during linguistic processing following ILAT. As reviewed in the introduction, one of the key factors for ILAT success at the clinical/behavioral level is the embedding of language into action and social interaction, realized in a card game context in group therapy sessions (Stahl et al., 2016). This therapy setting may be beneficial, in particular, for training the recruitment of domain-general attentional resources for language processing (Kiran & Thompson, 2019), as the social interactions and turn-taking activities fostered by this method require both focusing and division of the patients’ attention in social discourse (Difrancesco et al., 2012). In addition, previous evidence for an interplay of domain-general systems and ILAT outcome may also be seen in the results by Berthier et al. (2009). This study demonstrated additive beneficial effects of ILAT and pharmacological treatment with Memantine, a drug related to improvements in domaingeneral attention and episodic memory in dementia (Wesnes et al., 2015) and therapy of attention-deficit/hyperactivity disorder in pediatric populations (Findling et al., 2007). Facilitation of the recruitment of domain-general resources for language processing following ILAT may also explain the aforementioned carryover effect between language modalities in therapy and fMRI sessions, as those resources are recruited in the processing of both verbal and written processing. At the same time, we would like to highlight that the current findings do not allow for directly relating improved language competence to improved recruitment of attentional resources, as this domain was not tested behaviorally. Other interpretations, like an improved memory retrieval in language processing, are compatible with the current results as well (see Fridriksson et al., 2007; Musso et al., 1999). Furthermore, it is important to note that the current results are not informative on the issue whether the increased recruitment of domain-general systems following ILAT, as in the precuneus or the more extended DMN, has a causal role for clinical language improvement, or whether it is itself the consequence of other neural reorganization processes involved in aphasia recovery.

Our data did not reveal any specific relationship of language improvement to BOLD signal change in either right-hemispheric homotopic to left perisylvian language areas or in left-hemispheric perilesional regions, as they were reported in earlier studies addressing ILAT specifically (Breier et al., 2006, 2007, 2009; Lucchese et al., 2017; Meinzer et al., 2008; Mohr et al., 2014) or language recovery more generally (e.g., Crosson et al., 2005; Fridriksson et al., 2012; Heiss et al., 1999; Musso et al., 1999; Warburton et al., 1999; Saur et al., 2006). However, it may be that different neural circuits reorganized across patients or that the same ones reorganized to different degrees, depending on exact aphasia syndrome or lesion characteristics (Fridriksson et al., 2007). ROI-level, rather than voxel-level, analysis on the group level may be more appropriate to tolerate small variation in reorganization loci, as we tried here by focusing the current analysis on DMN and SN. This approach could, in principle, be applied to investigate the aforementioned contributions of left- and right-hemispheric areas to aphasia recovery, although this is beyond the scope of the current analysis.

An involvement of domain-general cortical systems in aphasia recovery may invite the idea to perform therapy solely targeting domain-general cognitive functions, including attention or memory, instead of performing classical aphasia therapy, focused on linguistic functions. We would like to stress, however, that the current analysis does not address or answer the question whether or not domain-general cognitive skill training is beneficial for aphasia therapy, as such training was not applied. It may well be that, for the present modulation of DMN activity to occur in a reading task, it is necessary to practice language use in variable tasks where specific lexical and grammatical items are specifically needed, as it is the case for the language games of ILAT. Furthermore, an improvement of domain-general cognitive functions independent of language processing following ILAT is at variance with our present data. The fMRI BOLD signal change correlation to AAT scores, from pre to post ILAT, was observed specifically for reading of proper words when contrasted against reading of hashmark strings, thus reflecting linguistic and not domain-general processing. In case of an entirely nonlinguistic, domain-general process, one would expect the processing of hashmark strings (baseline) to be affected in the very same way as the processing of meaningful words. If so, any non-linguistic domain-general processing advantage would be cancelled out in the critical comparison of words versus visual baseline. Therefore, it appears that any ILAT-related changes in domain-general cognitive networks occur specifically in the context of linguistic processing, possibly due to a more efficient recruitment of these general resources specifically for language. More clarity regarding this issue could come from further neuropsychological testing applied pre- and posttherapy, which could provide measures of attention or memory capacities outside a linguistic context, but such data are unfortunately not available for the patient sample presented here.

One caveat of the current study design is the absence of either a control group, which did not receive therapy, or a control scanning session of patients following a period without ILAT. Therefore, any effect on fMRI results could, in principle, resemble mere repetition or habituation effects. We believe, however, that, in light of the observed strong correlations to clinical language measures, this interpretation is at least unlikely to account for all fMRI results reported. Furthermore, previous magneto-encephalography investigations on the neural mechanisms underlying ILAT in chronic stroke patients (Breier et al., 2009) did not report significant differences of results following a 2-week interval without therapy in a single-word passive listening task. This paradigm was similar in design to the passive reading task applied in the current study, thus suggesting a substantial degree of stability in brain activation patterns for single-word processing across sessions in the absence of therapy. Direct evidence for the retest reliability of reading-related (rather than listening-related) fMRI results can also be seen in the results by Higgins et al. (2019), again suggesting that the differences between pre and post ILAT fMRI sessions are not driven by mere repetition effects.

In summary, the current data provide behavioral evidence for the efficiency of ILAT in people with chronic poststroke aphasia in improving clinical language test results. They also indicate that there are brain correlates of ILAT-related language performance improvements in the precuneus and in the DMN. As to our knowledge, these findings are the first to show an involvement of domain-general neural systems in language recovery following ILAT. Whether this involvement has a causal role in producing clinical language improvements or rather is just an index and “epiphenomenon” of language-relevant neural reorganization processes elsewhere instead can, however, not be concluded with certainty and is subject to future research.

## Supplementary Material

Supplementary Material

## Figures and Tables

**Table 1:** Sociodemographic, clinical, and therapy characteristics for individual patients included in the analysis.

Patient ID	Age	Sex	Months since stroke	Years of formal education	Diagnosis	Total ILAT duration (hr)	ILAT intensity (hr/week)
1	74	M	29	17	Wernicke’s aphasia	48	12
2	51	F	23	18	Amnesic aphasia	24	6.
3	74	M	24	19	Broca’s aphasia	24	6.
4	60	F	253	12	Broca’s aphasia	24	6.
5	33	F	12	13	Broca’s aphasia	48	12.
6	41	F	95	12	Broca’s aphasia	24	6.
7	39	F	45	11	Broca’s aphasia	24	6.
8	34	M	54	14	Broca’s aphasia	24	6.
9	82	F	197	13	Broca’s aphasia	48	12.
10	81	M	44	13	Broca’s aphasia	24	6.
11	51	F	178	13	Broca’s aphasia	48	12.
12	62	M	24	16	Global aphasia	25	12.5
13	33	M	18	13	Broca’s aphasia	25	12.5
14	60	F	84	22	Global aphasia	25	12.5
15	62	M	47	12	Nonclassifiable	25	12.5
16	58	M	25	19	Broca’s aphasia	25	12.5

*Note*. ILAT = intensive language-action therapy; M = male; F = female.

**Table 2:** Aachen Aphasia Test (AAT) mean *T* scores pretherapy (T1) and posttherapy (T2), as well as T2-T1 differences for individual patients.

Patient ID	AAT means T1	AAT means T2	AAT difference T2−T1
1	44.3	47	2.7
2	42	43	1
3	60.8	65.3	4.5
4	60.5	65.3	4.8
5	60	66.3	6.3
6	45.5	46.5	1
7	59.3	61.8	2.5
8	65.5	66.5	1
9	47.5	49.5	2
10	58	60	2
11	49.3	51.3	2
12	45.3	44.50	-0.8
13	51.3	54	2.8
14	59	59.25	0.3
15	54.5	54	-0.5
16	57	58.25	1.3

**Table 3:** Significant clusters for the effect of Aachen Aphasia Test (AAT) T2−T1 differences on T2−T1 signal change for the words versus visual baseline contrast, at a voxel-wise *p* <.005 and a minimum cluster size of *k* = 100.

Cluster location	Cluster size	MNI coordinates			t	p
		x	y	z		
Left precuneus	121	-10	-48	40	3.72	.0011

*Note*. MNI = Montréal Neurological Institute.
