# Supplementary material for: Increased Recruitment of Domain-General Neural Networks in Language Processing Following Intensive Language-Action Therapy: fMRI Evidence From People With Chronic Aphasia
Source: Am J Speech Lang Pathol. Author manuscript; Available in PMC 2022 Jul 28. (PMC7613191; doi:10.1044/2020_AJSLP-19-00150)
Supplement: Supplementary Material [file EMS151056-supplement-Supplementary_Material.pdf]

**Supplemental Material S1.** Supplemental figures and tables.

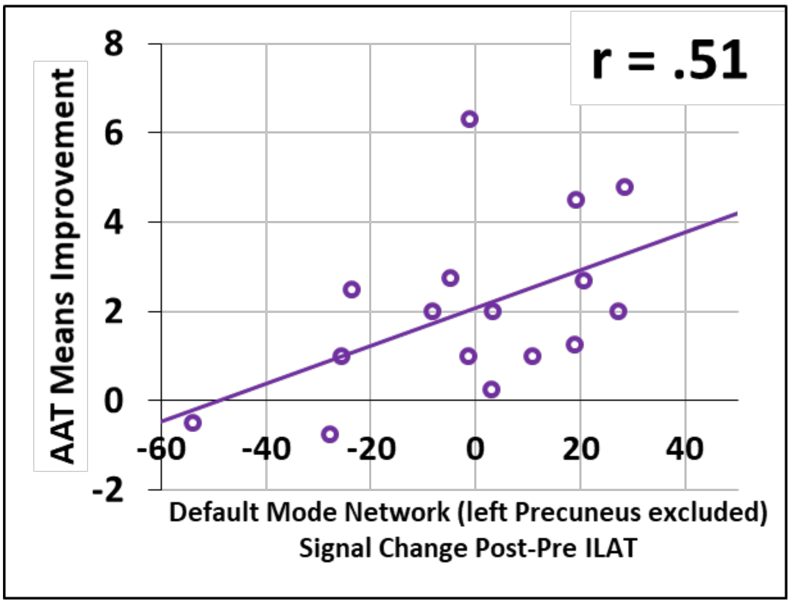


**Supplemental Figure S1:** Scatter plot of Words-vs-Hashmarks T2−T1 BOLD signal change and AAT results T2−T1 in the default mode network when the significant cluster in the left precuneus (see Figure 3) is excluded from ROI definition. Circles depict data from individual patients and the linear trendline is represented via the solid line.

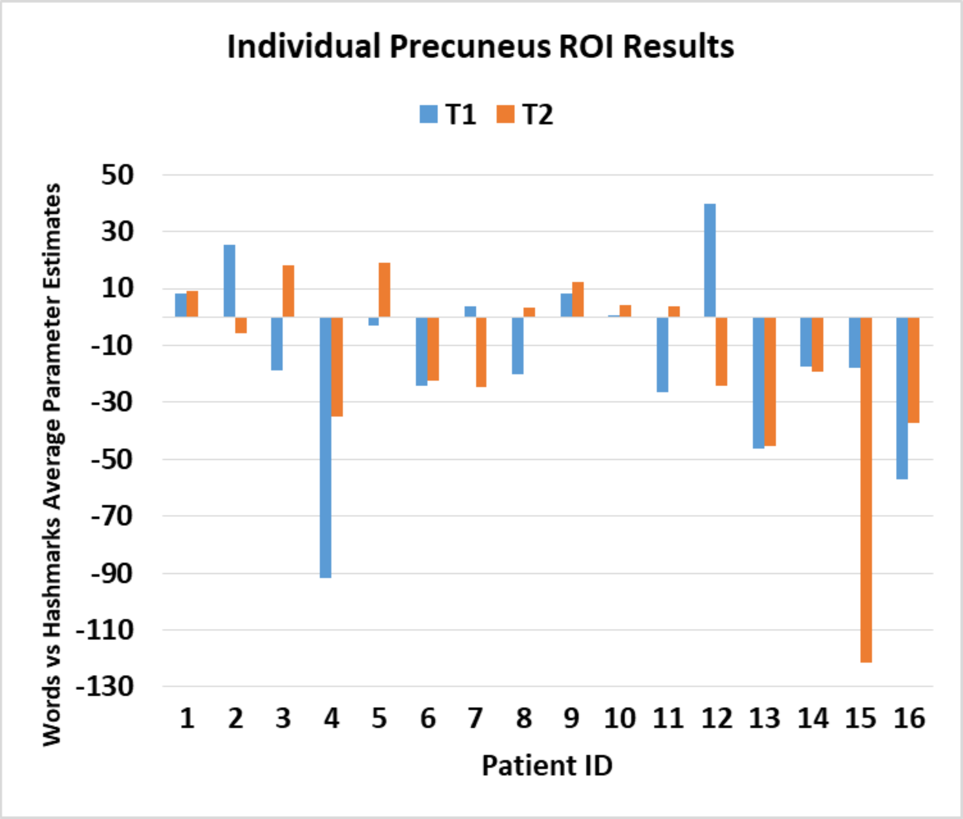


**Supplemental Figure S2:** Individual average parameter estimates for the contrast words vs. Hashmarks (visual baseline) in the precuneus ROI.

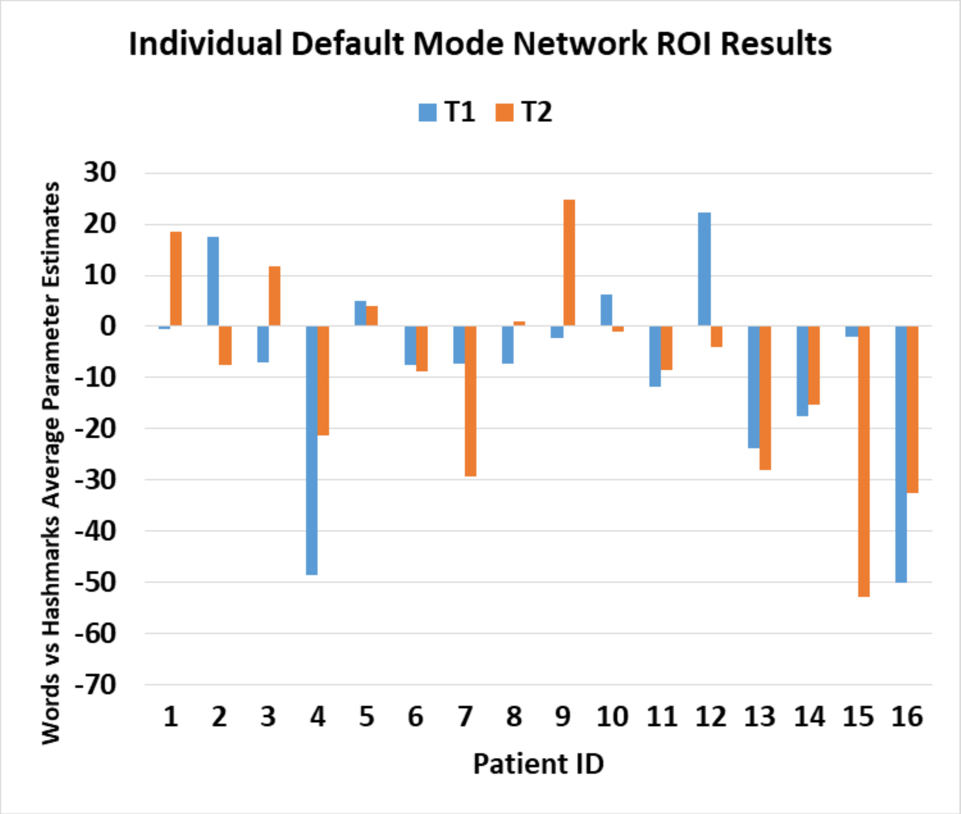


**Supplemental Figure S3:** Individual average parameter estimates for the contrast words vs. hashmarks (visual baseline) in the default mode network RO.

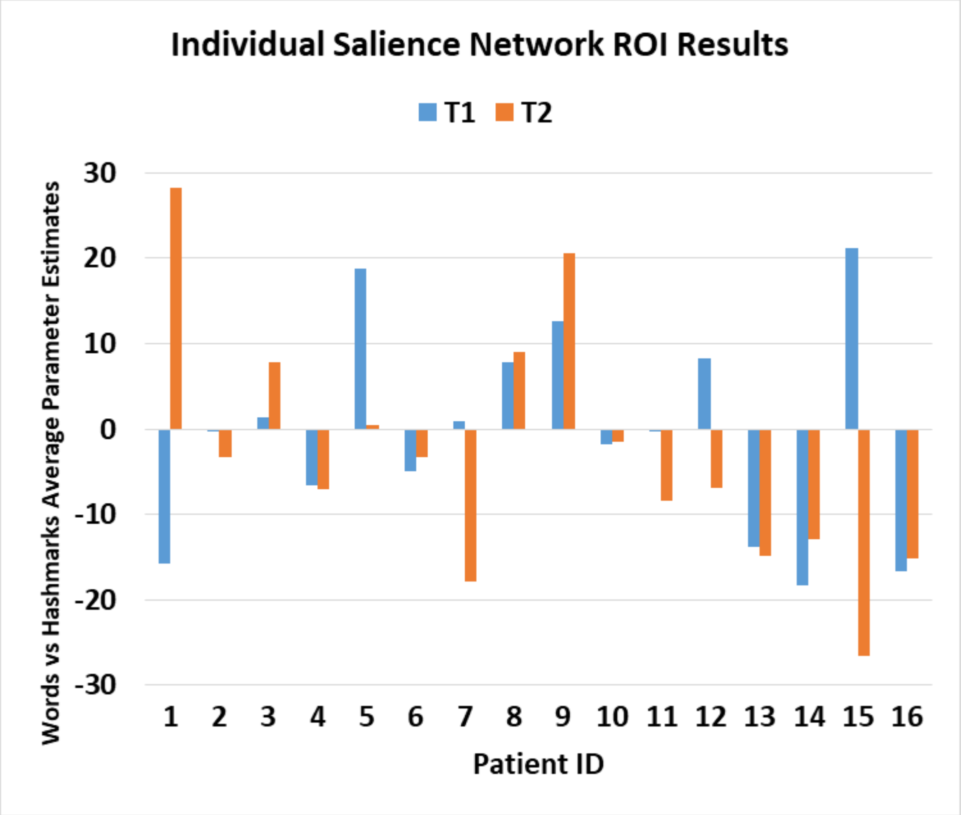


**Supplemental Figure S4:** Individual average parameter estimates for the contrast words vs. hashmarks (visual baseline) in the salience network ROI.

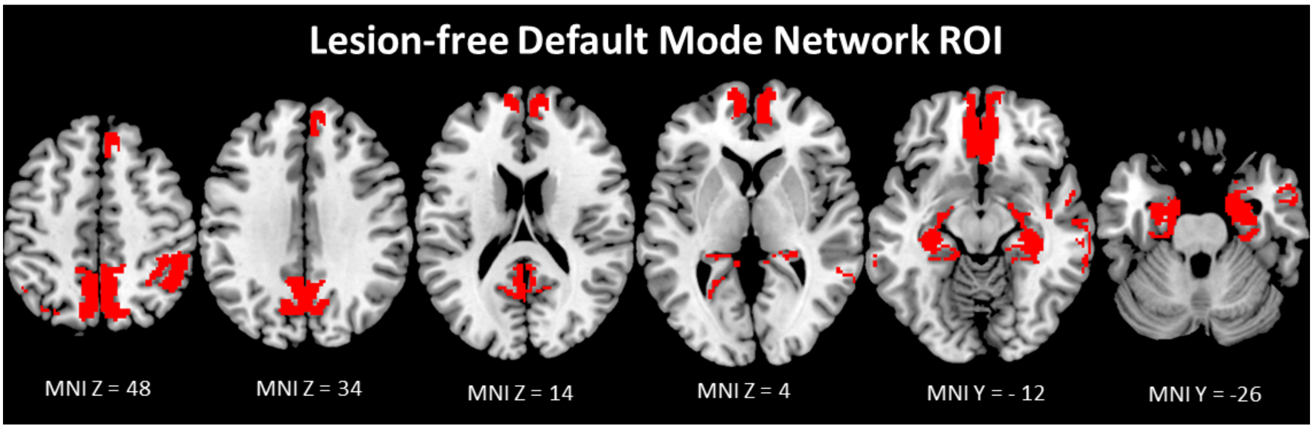


**Supplemental Figure S5:** Overview of the lesion-free default mode network ROI. Each horizontal slice is presented with the respective Z coordinate in MNI space.

**Supplemental Table S1:** Significant clusters for each time point for the words vs. hashmark (visual baseline) contrast, at a voxel-wise $p < .005$ and a minimum cluster size of $k = 100$.

| Contrast | Cluster location | Cluster Size | Peak MNI Coordinates x | y | z | t | p |
|---|---|---|---|---|---|---|---|
| T1 Words > Hashes | Right Supplementary Motor Area | 109 | 6 | 10 | 64 | 5.06 | < .001 |
| T2 Words > Hashes | Right Inferior Frontal Cortex Pars Orbitalis | 120 | 42 | 26 | -14 | 5.35 | < .001 |
| | | | 28 | 22 | -6 | 3.02 | < .001 |
| | Right Inferior Frontal Cortex, Pars Triangularis | 140 | 48 | 20 | 20 | 5.19 | < .001 |
| | | | 44 | 28 | 26 | 4.27 | < .001 |
| | Left Thalamus | 113 | -4 | -14 | 10 | 4.78 | < .001 |
| | Right Cerebelum | 210 | 10 | -80 | -24 | 3.3 | < .001 |
| | | | 16 | -74 | -22 | 3.09 | .001 |
| | | | 30 | -72 | -20 | 2.87 | .002 |

**Supplemental Table S2:** Significant clusters for the differences between time point on the words vs. hashmark (visual baseline) contrast, at a voxel-wise $p < .005$ and a minimum cluster size of $k = 40$.

| Contrast | Cluster location | Cluster Size | Peak MNI Coordinates x | y | z | t | p |
|---|---|---|---|---|---|---|---|
| T1-T2 Words > Hashes | *no significant clusters* | | | | | | |
| T2 -T1 Words > Hashes | Right Superior Occipital Cortex | 83 | 28 | -66 | 28 | 4.48 | < .001 |
| | Left Thalamus | 73 | -4 | -12 | 12 | 4.08 | < .001 |

**Supplemental Table S3:** Significant clusters for the effect of AAT T2−T1 differences on T2−T1 signal change for the words vs. visual baseline contrast using intensity or duration as additional covariates, at a voxel-wise $p < .005$ and a minimum cluster size of $k = 40$.

| Covariates | Cluster location | Cluster Size | Peak MNI Coordinates x | y | z | t | p |
|---|---|---|---|---|---|---|---|
| ILAT Intensity | Left Precuneus | 49 | -10 | -48 | 40 | 3.46 | .002 |
| ILAT Duration | Left Precuneus | 46 | -6 | -52 | 38 | 3.43 | .002 |

Please note that the cluster size threshold in Supplemental Tables S2 and S3 was considerably more liberal than in the remaining analyses for explorative purposes. As a cluster size of k = 100 was not reached in either analysis it was decreased to k = 40, following a stepwise lowering of the cluster size threshold by 10 voxels per step and then selecting a threshold 10 voxels lower than required for the emergence of first clusters in both analyses.
